# The Development and Evaluation of a Prediction Model for Kidney Transplant-Based *Pneumocystis carinii* Pneumonia Patients Based on Hematological Indicators

**DOI:** 10.3390/biomedicines12020366

**Published:** 2024-02-04

**Authors:** Long Zhang, Yiting Liu, Jilin Zou, Tianyu Wang, Haochong Hu, Yujie Zhou, Yifan Lu, Tao Qiu, Jiangqiao Zhou, Xiuheng Liu

**Affiliations:** 1 Department of Organ Transplantation, Renmin Hospital of Wuhan University, Wuhan 430060, China; rm002720@whu.edu.cn (L.Z.); lyt842303433@163.com (Y.L.); zoujilin@whu.edu.cn (J.Z.); wangty0510@whu.edu.cn (T.W.); hhc19970430@163.com (H.H.); zhouyujie0514@163.com (Y.Z.); yifanlu26@gmail.com (Y.L.); qiutao@whu.edu.cn (T.Q.); 2 Department of Urology, Renmin Hospital of Wuhan University, Wuhan 430060, China

**Keywords:** *Pneumocystis carinii* pneumonia (PCP), machine learning, prediction model, procalcitonin (PCT)

## Abstract

Background: This study aimed to develop a simple predictive model for early identification of the risk of adverse outcomes in kidney transplant-associated *Pneumocystis carinii* pneumonia (PCP) patients. Methods: This study encompassed 103 patients diagnosed with PCP, who received treatment at our hospital between 2018 and 2023. Among these participants, 20 were categorized as suffering from severe PCP, and, regrettably, 13 among them succumbed. Through the application of machine learning techniques and multivariate logistic regression analysis, two pivotal variables were discerned and subsequently integrated into a nomogram. The efficacy of the model was assessed via receiver operating characteristic (ROC) curves and calibration curves. Additionally, decision curve analysis (DCA) and a clinical impact curve (CIC) were employed to evaluate the clinical utility of the model. The Kaplan–Meier (KM) survival curves were utilized to ascertain the model’s aptitude for risk stratification. Results: Hematological markers, namely Procalcitonin (PCT) and C-reactive protein (CRP)-to-albumin ratio (CAR), were identified through machine learning and multivariate logistic regression. These variables were subsequently utilized to formulate a predictive model, presented in the form of a nomogram. The ROC curve exhibited commendable predictive accuracy in both internal validation (AUC = 0.861) and external validation (AUC = 0.896). Within a specific threshold probability range, both DCA and CIC demonstrated notable performance. Moreover, the KM survival curve further substantiated the nomogram’s efficacy in risk stratification. Conclusions: Based on hematological parameters, especially CAR and PCT, a simple nomogram was established to stratify prognostic risk in patients with renal transplant-related PCP.

## 1. Background

Renal transplantation stands as the most effective approach for treating end-stage renal disease. However, the prolonged administration of immunosuppressive agents following the procedure can compromise the immune system, rendering it susceptible to various opportunistic infections. Among these, PCP emerges as a prevalent complication following kidney transplantation. Its primary symptoms encompass fever, dry cough, progressive dyspnea, and hypoxemia. In severe cases, it may culminate in respiratory failure, posing a grave threat to patients’ lives [1,2]. Initially, PCP garnered attention primarily among individuals infected with the human immunodeficiency virus (HIV), subsequently extending to solid organ transplant recipients, hematopoietic stem cell transplant patients, and those undergoing treatment with corticosteroids [3,4]. The incidence of PCP has seen an upward trend owing to advances in organ transplantation, the expansion of the immunosuppressant-utilizing population, and the extension of postoperative survival rates [5,6,7].

Presently, all transplant centers have implemented comprehensive PCP prevention programs. Sulfamethoxazole/trimethoprim remains the preferred prophylactic agent for PCP prevention, with the recommendation that all kidney transplant recipients receive this medication within a specified timeframe after transplantation (typically from six to twelve months). Both daily and thrice-weekly dosing regimens have demonstrated equal effectiveness in preventing PCP infections during this prophylactic period. However, research indicates that PCP can still manifest up to one year post-surgery, despite six to twelve months of prophylactic treatment [8]. In comparison to HIV-positive patients, kidney transplant recipients afflicted with PCP generally experience a more aggressive disease course, with a mortality rate of up to 50% [9,10]. Following renal transplantation, PCP patients face a heightened risk of tracheal intubation, severe illness, early diagnostic challenges, and additional treatment complications such as renal insufficiency, all of which can lead to an unfavorable prognosis [11]. Therefore, effective prevention, early detection, and treatment of PCP in kidney transplant recipients, particularly severe cases, assume paramount importance in minimizing the incidence of severe disease and mortality rates.

Numerous studies have endeavored to predict the likelihood of PCP occurrence following organ transplantation. Research has revealed that persistent lymphocytopenia, cytomegalovirus infection (CMV), hypogammaglobulinemia, acute graft rejection, advanced age, renal insufficiency, HLA mismatch count exceeding 3, and a history of rituximab and anti-thymocyte globulin (ATG) use all constitute risk factors for PCP infection [10,12]. However, limited research has been conducted on predicting the onset and progression of severe PCP. Previous studies have indicated strong advantages of simple hematological markers such as c-reactive protein (CRP) and procalcitonin (PCT) in identifying PCP and assessing its severity in patients [13,14,15]. Additionally, routine hematological parameters hold value in predicting various complications post-kidney transplantation [16,17,18]. Hence, this study aims to establish an easily applicable prediction methodology for severe PCP, leveraging machine learning and multifactorial logistic regression screening methods. This approach will amalgamate some simple hematological test indicators from PCP patients upon admission, with the primary objective of facilitating early clinical intervention and treatment for kidney transplant-associated PCP patients in need.

## 2. Methods

### 2.1. Patient Groups

**Figure 1:**
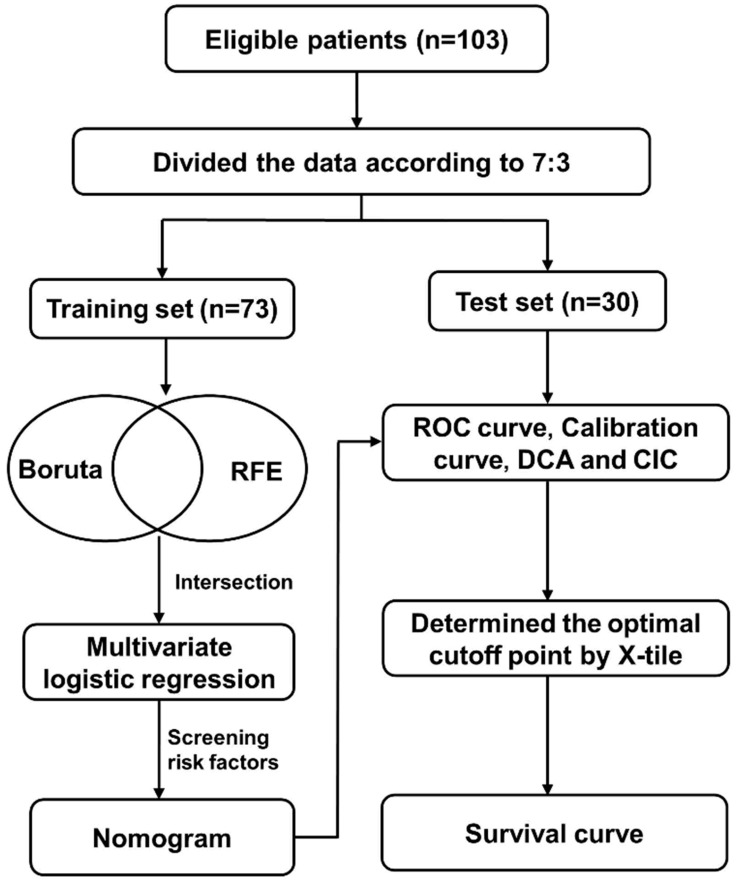
Flow diagram of the renal transplant-associated PCP patients in the training and test cohorts. PCP: *Pneumocystis carinii* pneumonia.

A total of 103 patients who had developed PCP following kidney transplantation at the Organ Transplantation Department of Renmin Hospital of Wuhan University between 2018 and 2023 were encompassed in this study. The inclusion criteria were as follows: (1) Metagenomic next-generation sequencing (mNGS) was employed to ascertain the existence of Pneumocystis by detecting microbial pathogens in the sputum or peripheral blood of kidney transplant recipients; (2) The maintenance of immunosuppressive agents, comprising mycophenolate mofetil + tacrolimus/cyclosporine + glucocorticoids. Patients were excluded if they met any of the subsequent criteria: (1) Had undergone relative kidney transplantation; (2) Had undergone multiple kidney transplantations or combined transplantations; (3) Possessed incomplete clinical data. The flow chart of the research design is shown in Figure 1. The study received approval from the Ethics Committee of Renmin Hospital of Wuhan University. We categorized PCP cases requiring admission to the intensive care unit (ICU) for treatment as severe PCP.

### 2.2. Clinical Data Collection

The collection of clinical data encompassed the following aspects: (I) Fundamental demographic information such as age, gender, and the duration following the transplant; (II) Clinical presentations observed at the time of hospitalization, including fever, dry cough, and progressive dyspnea; (III) Laboratory findings from the day of admission, consisting of the galactomannan (GM) test, PCT levels, white blood cell (WBC) count, serum creatinine (Scr) levels, neutrophil/lymphocyte ratio (NLR), glucose (Glu) levels, hemoglobin (Hb) levels, platelet count (Plt), blood urea nitrogen (BUN) levels, c-reactive protein/albumin ratio (CAR), presence of CMV infection (CMV DNA PCR titer ≥500 copies/mL in whole blood), and prior use of anti-thymocyte globulin (ATG) induction; (IV) Furthermore, the study tracked patient outcomes, which included the length of hospital stay, admission to the ICU, and mortality rates.

### 2.3. Treatment and Clinical Outcomes

All 103 patients ceased their immunosuppressive regimen and underwent treatment involving a blend of trimethoprim/sulfamethoxazole (TMP-SMZ) (TMP at a dosage of 15–20 mg/kg and SMZ at a dosage of 75–100 mg/kg, administered in three separate doses), along with caspofungin (50 mg/day, with an initial loading dose of 70 mg on the first day) to combat PCP. Furthermore, third-generation cephalosporins were administered as a prophylactic measure against bacterial infections. For patients with CMV infection, antiviral therapy employing ganciclovir was initiated, with drug dosages tailored to the initial stage of treatment. Methylprednisolone was utilized for the management of inflammation and fever, with dosage adjustments contingent upon the severity of the ailment (ranging from 40–120 mg/day, administered in two to three divided doses). Patient outcomes under scrutiny in our study encompassed ICU admission during hospitalization and in-hospital mortality. Most patients received oxygen therapy via nasal cannula or an oxygen mask throughout their hospitalization. In instances where the patients’ condition deteriorated beyond the capacity of the general ward, twenty individuals were transferred to the ICU for advanced treatment, ultimately resulting in thirteen fatalities.

### 2.4. Model Building, Validation, and Calibration

We established a nomogram model aimed at predicting the probability of ICU admission among patients with renal transplant-associated PCP. Our approach commenced by employing two machine learning techniques to identify the most pertinent variables within the training dataset. Subsequently, we conducted multifactorial logistic regression, selecting only those variables that exhibited statistical significance (*p* < 0.05) for integration into the logistic regression model, culminating in the creation of the nomogram. To refine our predictive accuracy, we utilized the variance inflation factor (VIF) to gauge the extent of multicollinearity among the selected variables. Variables with a VIF < 5 were deemed statistically insignificant. We further examined the relationship between the variables and the ultimate outcome using the spline function to assess linearity. We assessed the discriminative capacity of our model by constructing receiver operating characteristic (ROC) curves and computing the area under the curve (AUC). Calibration of the model was evaluated through Hosmer–Lemeshow tests, Brier scores, and calibration curves. DCA and CIC were plotted to evaluate the clinical usability of nomograms. Based on the resulting nomogram, we used X-Tiles to determine the optimal cut-off points and Kaplan–Meier curves to evaluate the ultimate risk stratification capability. 

### 2.5. Statistical Analysis

Continuous variables were calculated using an independent *t* test or Mann–Whitney U test and expressed as mean ± standard deviation (SD) or median (interquartile spacing). The categorical variables were calculated using the χ^2^ or Fisher exact test and expressed as numbers (percentages). R software (version 4.2.1, R Foundation for Statistical Computing, Vienna, Austria) and SPSS software (version 25.0, SPSS Inc., Chicago, IL, USA) were used for variable screening, data analysis, model construction, and graph rendering. X-Tile (version 3.6.1, Yale University, New Haven, CT, USA) software was used to determine the optimal cut-off point for the total score.

## 3. Results

### 3.1. Clinical Information

A total of 103 patients with renal transplant-related PCP participated in this study, of whom 20 (19.4%) were admitted to the ICU for treatment, and 13 (12.6%) of them died. The average age of the participants stood at 44.34 ± 10.13 years, with the majority (68%) being male. We divided the participants into a training set and a test set in a ratio of 7:3. Notably, no statistically significant distinctions were observed between the training and validation sets in terms of any of the characteristics, except for a statistical variance in the WBC count. Further details are provided in Table 1.

### 3.2. Variable Selection

**Figure 2:**
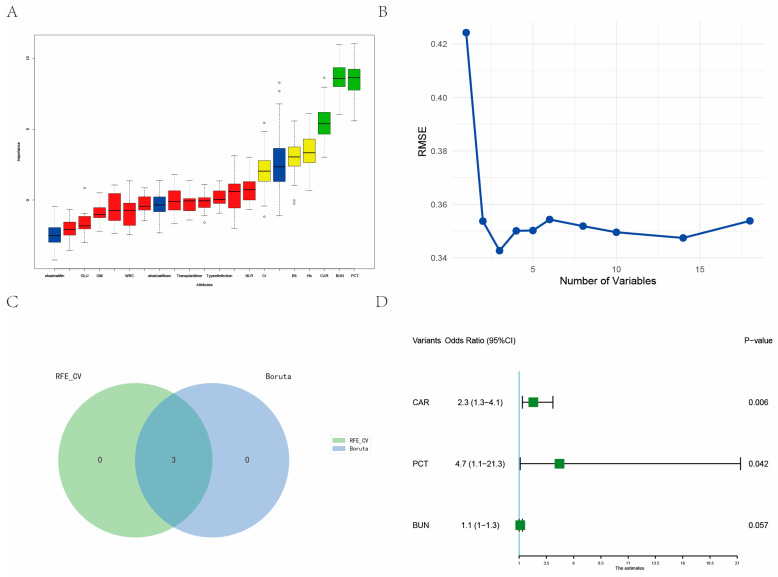
The variables were screened using machine learning and multi-factor logistic regression. (**A**) Boruta method filters variables; (**B**) RFE method to filter variables; (**C**) Venn diagram with the intersection of two machine learning methods; (**D**) Multivariate logistic regression screening variables. RFE: recursive feature elimination.

**Figure 3:**
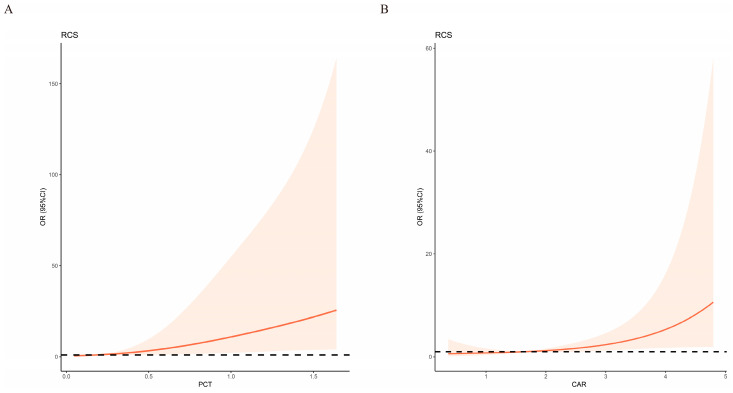
(**A**) The association between PCT and ICU was shown using RCS; (**B**) the association between CAR and ICU was shown using RCS. RCS: restricted cubic splines; PCT: procalcitonin; CAR: c-reactive protein/albumin ratio; ICU: intensive care unit.

To identify the most pertinent variables, we employed two machine learning techniques: Boruta (Figure 2A) and recursive feature elimination (RFE) (Figure 2B). Boruta is a machine learning algorithm based on random forests, designed to identify the most important features in a dataset. It assesses the significance of each feature by comparing its importance to randomly generated shadow features. If a feature’s importance is significantly higher than that of its corresponding randomly generated shadow feature, it is considered significant [19]. RFE is a recursive feature selection method that reduces the size of the feature set by iteratively building models and eliminating the least important features. In each iteration, RFE calculates feature weights and removes the feature with the lowest weight, continuing until the desired number of features is reached [20]. Through the intersection of these two methods, we identified PCT, CAR, and BUN as the optimal variables (Figure 2C). Subsequently, we conducted a multivariate logistic regression analysis involving these selected variables and generated a forest plot (Figure 2D). The outcomes of this analysis revealed that both PCT and CAR were independent risk factors for the occurrence of severe PCP. Further investigation into multicollinearity involving these two selected variables indicated a VIF of 1.017, signifying the absence of multicollinearity. We also constructed spline functions for PCT, CAR, and PCP severity separately. These analyses demonstrated that both PCT and CAR exhibited an approximately linear relationship with PCP severity (Figure 3A,B). Consequently, PCT and CAR can be directly represented as continuous variables.

### 3.3. Model Establishment and Validation

**Figure 4:**
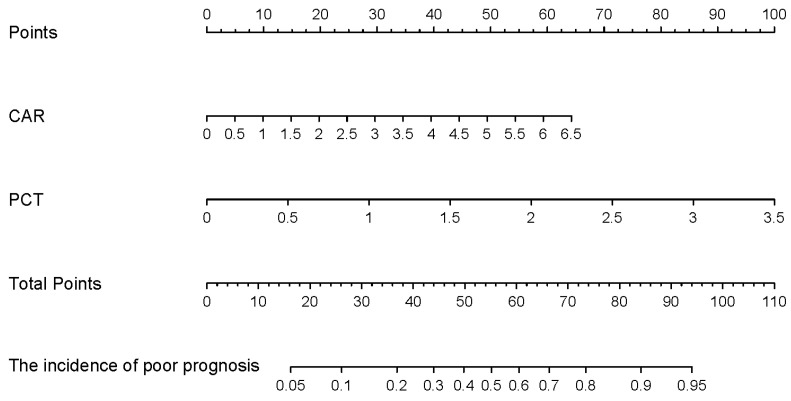
A nomogram for renal transplant-associated PCP patients and new risk stratification.

**Figure 5:**
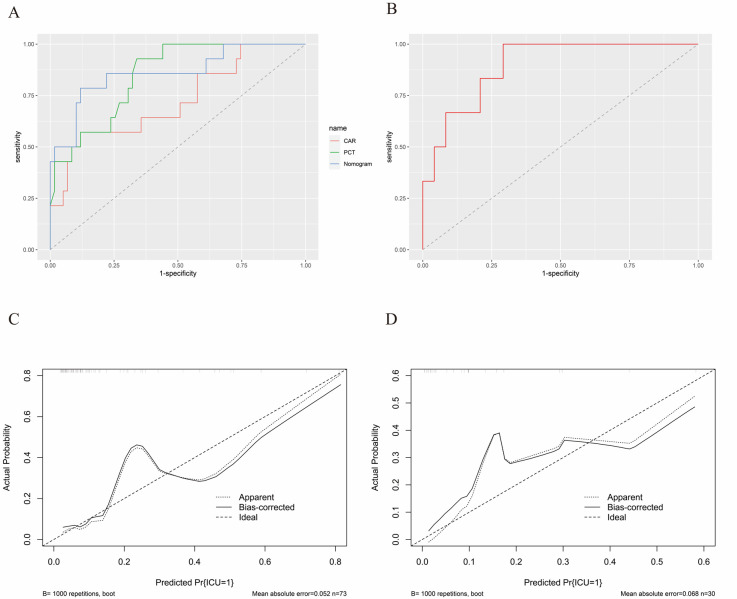
Model validation. (**A**) ROC Curve analysis of the predictive power for nomogram validation on the training set; (**B**) ROC Curve analysis of the predictive power for nomogram validation on the test set; (**C**) Calibration curve for nomogram validation on the training set; (**D**) Calibration curve for nomogram validation on the test set. ROC: receiver operating characteristic.

**Figure 6:**
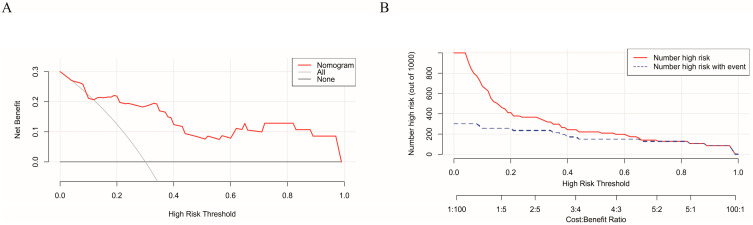
DCA and CIC curves were used to evaluate the clinical efficacy of the nomogram. (**A**) DCA curve. (**B**) CIC curve. DCA, decision curve analysis; CIC, clinical impact curve.

The two selected variables from the multivariate logistic regression model were utilized to refit the model, yielding the following predictive equation: Y = −4.1763 + 2.1636 × X1 + 0.7481 × X2, where X1 corresponds to PCT, and X2 pertains to CAR. The equation *p* = 1/(1 + exp(−Y)) was then employed to calculate the predictive value of the model for each patient. A direct association of PCT and CAR values to the points in the first row, as depicted in Figure 4, was used. These values were combined via addition to yield the total score. As illustrated in Figure 5A, the area under the ROC curve for the model in the training group was 0.861. Notably, the AUC of the model surpassed that of both PCT (AUC = 0.847) and CAR (AUC = 0.723). When compared to a solitary independent predictor, the nomogram exhibited superior predictive capability regarding the probability of renal transplant-related PCP necessitating ICU admission. The Hosmer–Lemeshow *p*-value for the training group was 0.741, indicating a well-fitting model. Additionally, we conducted external validation using data from the test group, yielding an area under the ROC curve of 0.896, as depicted in Figure 5B. Calibration curves derived from 1000 bootstrap samples demonstrated satisfactory calibration for both the model training group and the validation group, with Brier scores of 0.095 and 0.102, respectively (Figure 5C,D). To assess the clinical utility of the prognostic model, we applied DCA and plotted CIC, both of which showcased favorable performance across the threshold probability range (Figure 6A,B).

### 3.4. The Novel Risk Stratification System Based on a Nomogram

**Figure 7:**
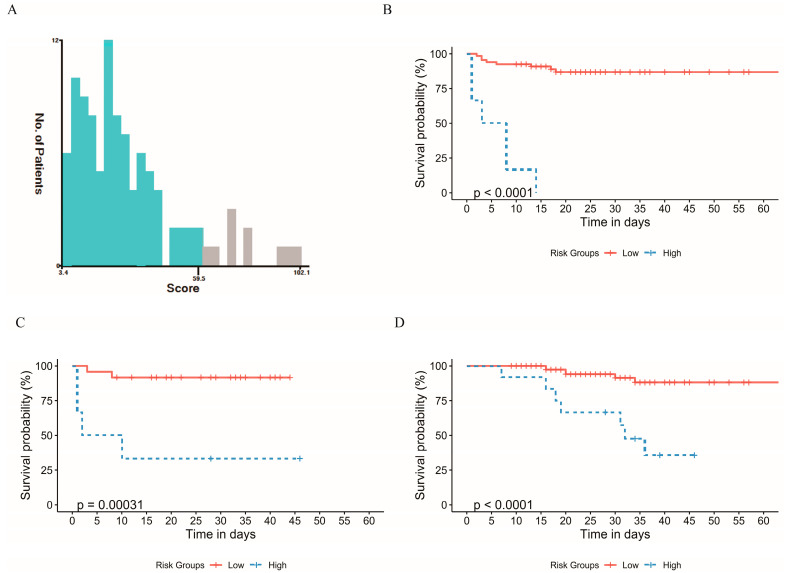
The novel risk stratification system. (**A**) The best cutoff for the total score achieved by X-Tile software was 59.5 (low risk: total score < 59.5 and high risk: total score ≥ 59.5). (**B**) The survival curve was used to assess the ability of the novel risk stratification system to distinguish PCP severe disease in the training cohort (**C**) The survival curve was used to assess the ability of the novel risk stratification system to distinguish PCP severe disease in the test cohort (**D**) The survival curve is used to assess the ability of the novel risk stratification system to distinguish deaths in the overall cohort.

We calculated and synthesized the total scores for all patients using the nomogram and then employed X-Tile software to categorize patients into two risk groups: low risk (total score < 59.5) and high risk (total score ≥ 59.5), as illustrated in Figure 7A. In both the training and validation cohorts, the Kaplan–Meier curves clearly demonstrated the efficacy of the new risk stratification system in distinguishing between the risk of ICU admission (Figure 7B,C). Furthermore, across the entire patient population, the Kaplan–Meier curve effectively stratified patients based on their risk of mortality (Figure 7D). Consequently, the new nomogram could be used to risk stratify patient outcomes.

## 4. Discussion

In individuals who have received a kidney transplant, the administration of immunosuppressive medications escalates the probability of acquiring postoperative PCP. The occurrence of PCP among kidney transplant recipients typically falls within a range of 1% to 10%, with the highest incidence being more commonly observed during the initial year following transplantation. PCP can manifest as fever, cough, dyspnea, and chest discomfort. In severe cases, respiratory insufficiency can occur, necessitating mechanical ventilation and admission to the ICU [21]. Due to the unfavorable prognosis associated with severe PCP, it is crucial to identify patients who are at risk of developing severe disease using limited clinical data.

Previous research has identified various risk factors associated with PCP, including HIV infection, organ transplantation, malignancies, certain inflammatory or rheumatic diseases, and related therapies that result in cell-mediated immune deficiency [22,23]. In kidney transplant patients, acute rejection and CMV infection have been identified as potential risk factors for PCP, and retrospective studies have shown that treatment with ATG during acute rejection may increase the risk of PCP [24]. Similarly, high doses of ATG and CMV infection have been found to be associated with an increased risk of PCP in kidney transplant patients [25]. Moreover, previous studies conducted by our group have demonstrated that the combination of PCP and CMV infection in kidney transplant patients is associated with a poorer prognosis than PCP alone [25]. A meta-analysis of risk factors for PCP in kidney transplant patients identified several clinical factors, such as HLA mismatch ≥3 at the time of PCP diagnosis, lymphocytopenia (absolute lymphocyte count ≤500 cells/mm^3^), rejection, CMV infection, and BK virus infection, as significantly increasing the risk of post-transplant PCP in renal transplant recipients. Additionally, the use of rituximab and polyclonal antibodies to treat rejection has been found to significantly increase the risk of PCP after transplantation [26]. 

Prior investigations predominantly emphasized the assessment of risk factors associated with PCP in kidney transplant recipients, with relatively few endeavors directed towards constructing predictive models capable of forecasting the progression to severe PCP and mortality in individuals afflicted with PCP following kidney transplantation. Therefore, this study focused on analyzing the results of relevant laboratory tests on the admission of renal transplant-related PCP patients, based on the results of the simplest blood tests to predict the occurrence of severe PCP, including CAR and PCT. Two machine learning methods were used for variable screening, and multivariate logistic regression was used to determine that both were independent risk factors for the development of renal transplant-related PCP into severe disease.

CAR serves as a valuable clinical indicator, offering insights into both the inflammatory response and nutritional status in patients with a comprehensive range of conditions. It has been widely employed to forecast the prognosis of various infectious diseases due to the convenience of obtaining test results [27,28,29,30,31,32,33,34,35]. CAR encompass two key components: CRP and ALB. CRP, a protein synthesized by the liver, plays a pivotal role in the body’s defense mechanisms in response to inflammation or infection. Following an inflammatory reaction, CRP levels significantly surge in the bloodstream. This makes it a nonspecific marker of inflammation, capable of indicating the presence of infection, tissue damage, autoimmune disorders, or even cancer [36,37,38,39,40]. Additionally, ALB levels at the time of admission can offer insights into the nutritional status of patients, which may, in turn, influence the prognosis of individuals with PCP [41,42,43]. While the utility of CAR in predicting adverse outcomes in patients afflicted with COVID-19, severe sepsis, or septic shock has been well documented [44,45,46,47], no prior investigations had delved into its prognostic potential in the context of renal transplant-associated PCP. The findings of this study have unveiled CAR as an independent risk factor for the severity of renal transplant-related PCP. Furthermore, spline function analysis demonstrated an approximate linear positive correlation between CAR and the probability of experiencing severe renal transplant-related PCP. Consequently, the measurement of admission CAR can serve as an effective means of gauging patient prognosis.

PCT, serving as a precursor to calcitonin, plays a crucial role in systemic responses elicited by circulating endotoxins and inflammatory cytokines. It has garnered recognition as a dependable marker of infection [48,49]. Previous investigations have demonstrated its utility in forecasting the prognosis of diverse patient groups, including those with conditions such as hospital or community-acquired pneumonia, cervical cancer, hospitalized children, aneurysmal subarachnoid hemorrhage, and cardiac surgery [50,51,52,53,54]. PCT often exhibits greater sensitivity to bacterial infections compared to other markers of the inflammatory response [55]. While prior studies have harnessed PCT to predict the prognosis of patients with COVID-19 and community-acquired pneumonia, demonstrating its efficacy [56,57,58,59,60], limited research had explored its potential in forecasting the prognosis of kidney transplant-related PCP. However, this study has uncovered PCT as an independent risk factor for the severity of renal transplant-related PCP. Moreover, spline function analysis has delineated an approximate linear positive correlation between PCT levels and the likelihood of experiencing severe renal transplant-related PCP. Hence, assessing PCT levels upon admission stands as an effective means to forecast the prognosis of patients grappling with this condition.

In this study, we developed an interpretable nomogram utilizing machine learning and multivariate logistic regression to predict the prognosis of renal transplant-related PCP. By combining CAR and PCT, we established a prediction model that outperforms reliance on a single clinical indicator. This model achieved prediction probabilities of 0.861 and 0.896 in the training group and the test group, respectively. Additionally, our redefined scoring system, based on the two indicators with a cutoff value of 59.5, effectively stratified the risk of prognosis related to PCP after kidney transplantation. It not only identifies the risk of severe PCP but also predicts the risk of in-hospital mortality. Clinicians can easily obtain the patient’s total score by mapping the CAR and PCT values from the nomogram’s first row and summing them up. Patients with scores exceeding 59.5 are classified into the high-risk group. Identifying patients prone to severe disease allows for targeted interventions such as enhanced nutritional support, adequate supplementation of human blood albumin, and intensified respiratory support like high-flow oxygen administration. Moreover, escalated doses of co-trimoxazole and caspofungin are administered, while hormone doses are adjusted appropriately. If the patient’s financial condition permits, he or she can be transferred to the ICU as soon as possible for individual care and better respiratory support. Therefore, the predictive model, based on direct blood testing indicators, has the potential to provide valuable assistance to clinicians in the early detection of severe cases. However, it is important to note that this study was conducted at a single center and involved a relatively small sample size. To enhance the robustness and generalizability of these findings, future endeavors should consider conducting multi-center studies with an extended follow-up period.

## Figures and Tables

**Table 1:** Baseline demographic and clinical laboratory examination characteristics of all patients (training and test groups).

	All, n = 103	Training, n = 73	Test, n = 30	*p*-Value
Age, yr	44.34 ± 10.13	44.40 ± 9.27	44.20 ± 12.16	0.929
Male, n	70 (68.0%)	51 (69.9%)	19 (63.3%)	0.68
Post-transplant time, months	8.00 [5.00, 14.00]	8.00 [5.00, 15.00]	7.50 [5.00, 10.75]	0.563
Fever, n	77 (74.8%)	54 (74.0%)	23 (76.7%)	0.971
Dry cough, n	30 (29.1%)	25 (34.2%)	5 (16.7%)	0.122
Progressive dyspnea, n	60 (58.3%)	41 (56.2%)	19 (63.3%)	0.652
GM test number	478.90 [209.00, 600.00]	491.00 [240.52, 600.00]	429.05 [145.43, 600.00]	0.339
PCT, ng/mL	0.20 [0.09, 0.34]	0.18 [0.10, 0.29]	0.23 [0.09, 0.54]	0.256
CAR, 1 × 10^−3^	1.68 [0.93, 2.61]	1.64 [0.95, 2.40]	2.19 [0.77, 2.71]	0.604
Hb, g/L	109.64 ± 19.01	110.03 ± 19.94	108.70 ± 16.82	0.749
Plt, 1 × 10^9^	211.00 [167.00, 255.00]	210.00 [152.00, 254.00]	217.00 [191.50, 256.25]	0.336
BUN, mg/dL	11.70 [8.78, 18.57]	12.00 [8.76, 18.40]	11.55 [10.01, 18.54]	0.816
Glu, mmol/L	6.30 [5.30, 8.37]	6.36 [5.40, 8.42]	5.96 [5.20, 7.70]	0.418
WBC, 1 × 10^9^	8.15 [5.12, 10.16]	6.90 [4.81, 9.80]	9.56 [7.45, 13.11]	0.001
Scr, μmol/L	150.00 [122.00, 189.50]	151.00 [124.00, 189.00]	146.00 [118.00, 192.50]	0.865
NLR	10.33 [7.14, 18.03]	9.76 [5.84, 17.86]	12.57 [8.80, 21.89]	0.115
ATG dose, mg	0.00 [0.00, 75.00]	0.00 [0.00, 75.00]	0.00 [0.00, 75.00]	0.336
CMV infection, n	43 (41.7%)	29 (39.7%)	14 (46.7%)	0.668
ICU, n	20 (19.4%)	14 (19.2%)	6 (20.0%)	1
Death, n	13 (12.6%)	8 (11.0%)	5 (16.7%)	0.641
Duration of hospitalization, days	27.00 [18.00, 35.50]	26.00 [18.00, 36.00]	28.50 [19.00, 34.75]	0.611

GM test: galactomannan test; PCT: procalcitonin; CAR: c-reactive protein/albumin ratio; Hb: Hemoglobin; Plt: platelet count; BUN: blood urea nitrogen; Glu: glucose; WBC: white blood cell; Scr: serum creatinine; NLR: neutrocyte/lymphocyte ratio; ATG: antihuman thymocyte globulin; CMV: cytomegalovirus; ICU: intensive care unit.

## Data Availability

The datasets used and/or analyzed during this current study are available from the corresponding author on reasonable request.
